# Perturbation of the Actin Cytoskeleton in Human Hepatoma Cells Influences Interleukin-6 (IL-6) Signaling, but Not Soluble IL-6 Receptor Generation or NF-κB Activation

**DOI:** 10.3390/ijms22137171

**Published:** 2021-07-02

**Authors:** Elizabeta Georgieva, Stefan L. Leber, Cora Wex, Christoph Garbers

**Affiliations:** 1 Department of Pathology, Medical Faculty, Otto-von-Guericke University Magdeburg, 39120 Magdeburg, Germany; elizabeta.b.georgieva@gmail.com; 2 Division of Neuroradiology, Vascular & Interventional Radiology, Department of Radiology, Medical University of Graz, 8036 Graz, Austria; stefan.leber@medunigraz.at; 3 Department of General, Visceral, Vascular and Transplantation Surgery, Medical Faculty, Otto-von-Guericke University Magdeburg, 39120 Magdeburg, Germany; cora.wex@med.ovgu.de

**Keywords:** interleukin-6, interleukin-6 receptor, NF-κB, actin, STAT3

## Abstract

The transcription factor nuclear factor-kappa B (NF-κB) is critically involved in inflammation and cancer development. Activation of NF-κB induces the expression and release of several pro-inflammatory proteins, which include the cytokine interleukin-6 (IL-6). Perturbation of the actin cytoskeleton has been previously shown to activate NF-κB signaling. In this study, we analyze the influence of different compounds that modulate the actin cytoskeleton on NF-κB activation, IL-6 signaling and the proteolytic generation of the soluble IL-6 receptor (sIL-6R) in human hepatoma cells. We show that perturbation of the actin cytoskeleton is not sufficient to induce NF-κB activation and IL-6 secretion. However, perturbation of the actin cytoskeleton reduces IL-6-induced activation of the transcription factor STAT3 in Hep3B cells. In contrast, IL-6R proteolysis by the metalloprotease ADAM10 did not depend upon the integrity of the actin cytoskeleton. In summary, we uncover a previously unknown function of the actin cytoskeleton in IL-6-mediated signal transduction in Hep3B cells.

## 1. Introduction

Interleukin-6 (IL-6) is the most famous member of the IL-6 family of cytokines [1]. It is secreted from several different cell types, including immune cells like neutrophils and macrophages [2]. Expression of IL-6 is induced in response to different extracellular cues, e.g., activation of Toll-like receptor 4 (TLR4) by lipopolysaccharide (LPS) [3]. One of the key proteins controlling IL-6 expression is the transcription factor nuclear factor-kappa B (NF-κB) [3,4]. In order to activate cells, IL-6 binds to the membrane-bound non-signaling IL-6 α-receptor (IL-6R). The IL-6/IL-6R complex induces homodimerization of the signal-transducing β-receptor gp130 and finally cumulates in the activation of several intracellular signaling cascades, most notably the Jak/STAT pathway [2,5]. Elevated serum levels of IL-6 are found in many inflammatory diseases, and the blockade of IL-6 signaling with antibodies targeting either IL-6 or the IL-6R is used successfully in the clinic, e.g., for patients with rheumatoid arthritis [6].

In addition to classic signaling via the membrane-bound IL-6R, IL-6 can also bind to soluble forms of the IL-6R (sIL-6R). While most soluble cytokine receptors are antagonists [7], the sIL-6R/IL-6 complexes are biologically active and bind to and activate gp130 homodimers on virtually all cells due to the ubiquitous expression of gp130. This so-called trans-signaling is initiated by proteolytic cleavage of the membrane-bound precursor and can be executed by different proteases, although the two metalloproteases ADAM10 and ADAM17 appear to be the most important IL-6R sheddases [8,9,10,11,12]. Specific inhibition of IL-6 trans-signaling by the compound olamkicept, which is the extracellular part of gp130 dimerized via the Fc portion of a human IgG antibody, is currently in clinical development and has recently been evaluated in a phase IIa clinical trial in patients with inflammatory bowel disease [13].

Previous studies have shown that NF-κB signaling and the actin cytoskeleton are closely linked and that the stabilization of actin filaments prevents nuclear translocation of NF-κB [14]. Actin dynamics can be modulated by several naturally occurring compounds, including cytochalasins and jasplakinolide [15], and this has been demonstrated to lead to the activation of NF-κB signaling in myelomonocytic [16] and intestinal epithelial cells [17]. However, whether this is a common mechanism that occurs in all cell types has not been investigated.

In the present study, we analyzed a possible role of the actin cytoskeleton on NF-κB activation, IL-6 signaling and sIL-6R generation. We show that NF-κB activation and sIL-6R generation are independent of the actin cytoskeleton, but that IL-6 signaling in Hep3B cells is reduced upon perturbation of actin.

## 2. Results

### 2.1. Short-Term Perturbation of the Actin Cytoskeleton Does Not Activate NF-κB

**Figure 1:**
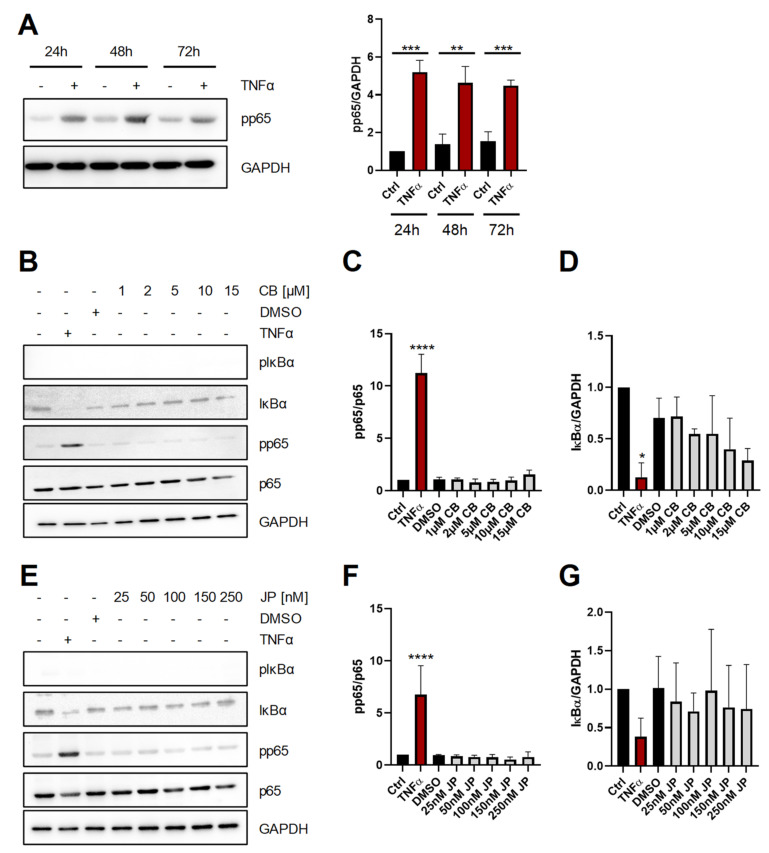
Short-term perturbation of the actin cytoskeleton does not activate NF-κB. (**A**) Stimulation of Hep3B cells with TNFα (100 U/mL) for 30 min 24 h, 48 h and 72 h after seeding. Representative immunoblot of phospho (p)p65 expression. GAPDH was visualized to verify equal loading. Data present the quantification of pp65 expression normalized to GAPDH. Data are shown as means ± SD of three independent experiments. Statistical significance was determined using two-tailed unpaired *t* tests (**: *p* < 0.01; ***: *p* < 0.001). (**B**) Stimulation of Hep3B with TNFα and cytochalasin B at increasing concentrations for 15 min. Representative immunoblots for pIκBα, IκBα pp65, p65 and GAPDH. (**C**,**D**) Quantification of expression of (**C**) pp65/p65 and (**D**) IκBα/GAPDH. Data are shown as means ± SD of three independent experiments. (**E–G**) The experiments were performed as described for panels B-D, but cells were pre-incubated with different amounts of jasplakinolide instead of CB. Statistical significance was determined using one-way ANOVA with Dunnett’s multiple comparisons test (all treated samples were compared to the DMSO-treated cells; *: *p* < 0.05; ****: *p* < 0.001; all other samples were not significantly different). CB: cytochalasin B; JP: jasplakinolide.

In order to analyze the influence of actin modulating compounds on NF-κB activation, we first verified that we were able to detect NF-κB activation in Hep3B cells and stimulated them 24 h, 48 h and 72 h after seeding with 100 U/mL TNFα for 30 min. As shown in Figure 1A, TNFα induced robust and significant phosphorylation of the NF-κB p65 subunit at all time points investigated (24 h: 5.2 ± 0.6-fold increase; 48 h: 4.6 ± 0.9-fold increase; 72 h: 4.5 ± 0.3-fold increase, all compared to the corresponding unstimulated control). We then added cytochalasin B (CB), which is a cell-permeable compound that inhibits actin filament network formation [15], for 15 min to the cells. In contrast to TNFα stimulation, increasing amounts of CB (1–15 µM) did not increase phosphorylation of p65 or induce degradation of IκBα, both of which are established indicators of NF-κB activation (Figure 1B–D). Next, we treated Hep3B cells for 15 min with jasplakinolide (JP), a macrocyclic peptide that strongly induces actin polymerization by the stimulation of actin filament nucleation [15]. As seen for CB, also treatment with increasing amounts of JP (25–250 nM) did not activate NF-κB as judged by phosphorylation of p65 and degradation of IκBα, which was again only observed when cells were treated with TNFα (Figure 1E–G). In conclusion, these data indicate that short-term perturbation of the actin cytoskeleton is insufficient to activate the NF-κB pathway.

### 2.2. Long-Term Perturbation of the Actin Cytoskeleton Does Not Activate NF-κB or Induce IL-6 Production

**Figure 2:**
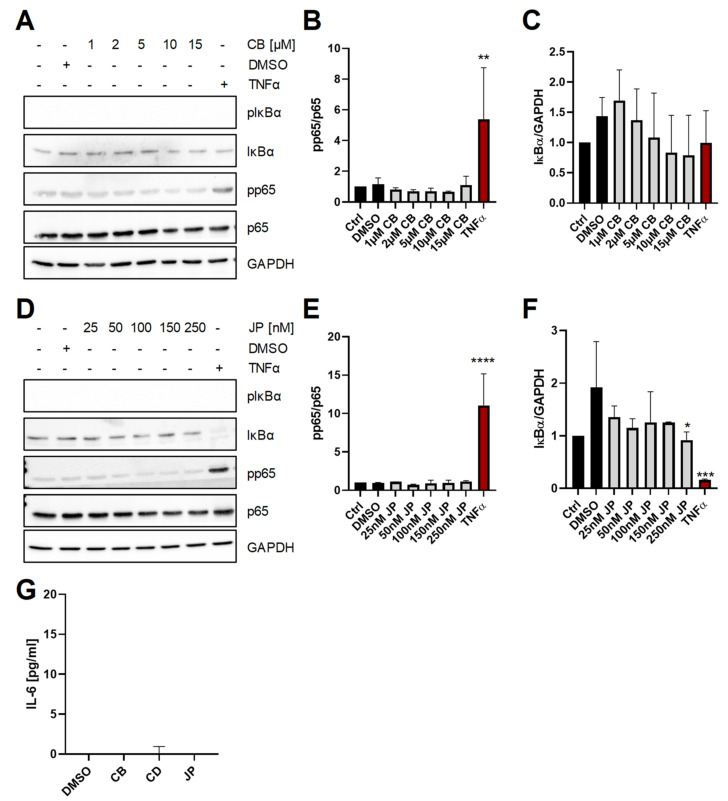
Long-term perturbation of the actin cytoskeleton does not activate NF-κB or induce IL-6 secretion. (**A**–**F**) The experiments were conducted as described in the legend to Figure 1, but the cells were stimulated for 120 min with the compounds indicated. Data are shown as means ± SD of two or three independent experiments. Statistical significance was determined using one-way ANOVA with Dunnett’s multiple comparisons test (all treated samples were compared to the DMSO-treated cells; *: *p* < 0.05; **: *p* < 0.01; ***: *p* < 0.001; ****: *p* < 0.001; all other samples were not significantly different). (**G**) ELISA quantification of IL-6 in the culture supernatant after pre-treatment of Hep3B cells with cytochalasin B (15 µM), cytochalasin D (1 µg) and jasplakinolide (0.25 µM) for 120 min. Data are shown as means ± SD of three independent experiments. Statistical significance was determined using one-way ANOVA with Dunnett’s multiple comparisons test and revealed no differences. CB: cytochalasin B; CD: cytochalasin D; JP: jasplakinolide.

Because we wanted to ensure that the results are not due to the rather short exposure time of the cells to the two compounds, we performed similar experiments in which we stimulated Hep3B cells for 120 min. As seen before, increasing amounts of CB (1–15µM) did neither increase phosphorylation of p65 nor induce degradation of IκBα, while stimulation with TNFα activated NF-κB signaling (Figure 2A–C). Similarly, treatment of Hep3B cells for 120 min with increasing amounts of JP (25–250 nM) did not activate NF-κB, because neither phosphorylation of p65 and nor substantial degradation of IκBα could be detected as opposed to cells stimulated with TNFα (Figure 2D–F). Because *IL6* is a well-known target gene of NF-κB [2], we also determined secretion of IL-6 into the cell culture supernatant after stimulation of Hep3B cells with CB, the related compound cytochalasin D (CD) or JP for 120 min. In agreement with our previous results, we could not detect any IL-6 in the cell culture supernatants (Figure 2G), further substantiating the lack of NF-κB activation even after prolonged perturbation of the actin cytoskeleton.

### 2.3. Perturbation of the Actin Cytoskeleton Interferes with STAT3 Signaling in Hep3B Cells

**Figure 3:**
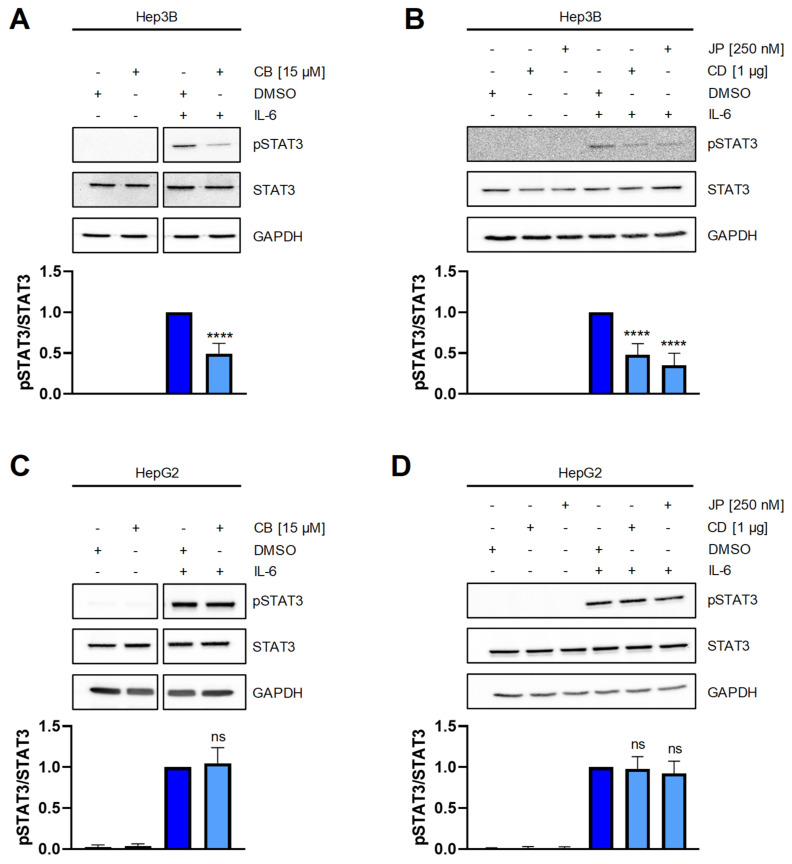
Perturbation of the actin cytoskeleton interferes with STAT3 signaling in Hep3B cells. (**A**,**B**) Hep3B cells were pretreated with cytochalasin B (15 µM), cytochalasin D (1 µg), jasplakinolide (250 nM) or DMSO (0.1%) as a control for 120 min. IL-6 (10 ng/mL) was added for 15min in order to activate STAT3 signaling. Representative western blot images from one experiment are shown, the quantitative data are shown as means ± SD of three independent experiments. (**C**,**D**) The experiments were performed as described for panels A and B, but HepG2 cells were used. Representative western blot images from one experiment are shown, the quantitative data are shown as means ± SD of three independent experiments. Statistical significance was determined using one-way ANOVA with Sidaks’s multiple comparisons test (cells pre-treated with actin-modulating compounds and stimulated with IL-6 were compared to IL-6-stimulated cells only; ****: *p* < 0.001; ns = no significant difference CB: cytochalasin B; CD: cytochalasin D; JP: jasplakinolide.

Having shown that perturbation of the actin cytoskeleton does not activate NF-κB signaling and subsequently does not induce IL-6 release, we wondered whether it might influence IL-6 signaling. In order to analyze this, we determined phosphorylation at tyrosine residue 705 of the transcription factor STAT3 on cells activated by IL-6 that had been pre-treated with different actin-modulating compounds. As shown in Figure 3A, IL-6 induced robust STAT3 phosphorylation of Hep3B cells, which was significantly reduced when cells were pre-treated with CB. Similarly, we observed reduced STAT3 phosphorylation when Hep3B cells were pre-treated with CD or JP (Figure 3B). In order to determine whether this effect was restricted to Hep3B cells or could be also seen in other hepatoma cell lines, we performed similar experiments with HepG2 cells, which we have previously used to analyze IL-6 signaling [18]. Interestingly, we could not detect any influence of CB, CD or JP on STAT3 phoshorylation following IL-6 stimulation (Figure 3C,D). In conclusion, perturbation of the actin cytoskeleton influences IL-6 signaling in Hep3B, but not HepG2 cells.

### 2.4. Perturbation of the Actin Cytoskeleton Does Not Influence IL-6R Proteolysis by ADAM10

**Figure 4:**
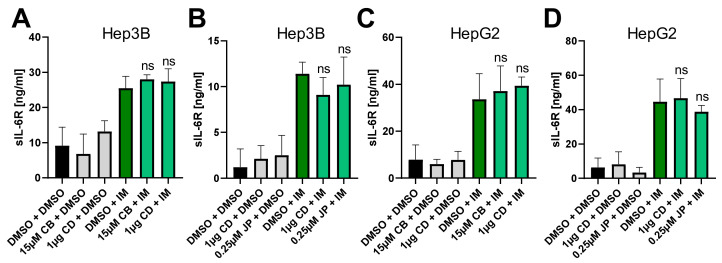
Perturbation of the actin cytoskeleton does not influence IL-6R proteolysis by ADAM10. (**A**,**B**) ELISA quantification of sIL-6R in culture supernatant after pretreatment of Hep3B cells with (**A**) cytochalasin B (15 µM) and D (1 µg) or (**B**) cytochalasin D (1 µg) and jasplakinolide (0.25 µM) for 120 min. Proteolysis by ADAM10 was activated via ionomycin (1 µM) for 60 min. Data are shown as means ± SD of three independent experiments. (**C**,**D**) The experiments were performed as described for panels A and B, but HepG2 cells were used. Data are shown as means ± SD of three independent experiments. Statistical significance was determined using one-way ANOVA with Sidaks’s multiple comparisons test (cells pre-treated with actin-modulating compounds and stimulated with ionomycin were compared to ionomycin-stimulated cells only). ns = no significant difference; CB: cytochalasin B; CD: cytochalasin D; JP: jasplakinolide; IM: ionomycin.

Proteolysis of the IL-6R by the metalloproteases ADAM10 and ADAM17 is a crucial aspect of IL-6 biology, as it constitutes the molecular switch between IL-6 classic and trans-signaling [19,20]. Therefore, we analyzed IL-6R proteolysis by ADAM10 of cells endogenously expressing IL-6R following pre-incubation with actin-modulating compounds. IL-6R is constitutively shed by ADAM10, but this can also be further induced by the ionophor ionomycin [11]. Neither CB nor CD influenced constitutive or induced IL-6R proteolysis by ADAM10 in Hep3B cells (Figure 4A), and the same was true for CD and JP (Figure 4B). We also verified these results in HepG2 cells, where we also did not detect any influence on sIL-6R generation by the three compounds (Figure 4C,D). We concluded from these results that ADAM10-mediated proteolysis of the IL-6R occurs independently of the actin cytoskeleton.

## 3. Discussion

The crucial role of cytokines like TNFα and IL-6 in numerous inflammatory diseases has made these proteins important therapeutic targets [6,21]. Designer proteins and antibodies have been generated to block the biological activities of these cytokines and are nowadays used to treat patients with e.g., rheumatoid arthritis or inflammatory bowel disease. Interestingly, the expression of both cytokines is controlled by the transcription factor NF-κB, which for this reason is itself an interesting point for intervention. However, although several approaches have been conducted to target NF-κB directly and there are several encouraging studies in animal models, no inhibitors have made their way into the clinic yet [4].

In this study, we analyzed whether NF-κB signaling in human hepatoma cells can be activated when the actin cytoskeleton is modulated by different chemical compounds. Previous studies have shown that the stabilization of actin filaments prevents nuclear translocation of NF-κB [14] and that its perturbation leads to the activation of NF-κB signaling in myelomonocytic [16] and intestinal epithelial cells [17]. However, our data show that this mechanism does not occur in human hepatoma cell lines. Moreover, the treatment of different cell lines with the microtubule-disrupting agent nocodazole also led to activation of NF-κB [22,23], and it is tempting to speculate that it might have a similar effect on the IL-6 system as demonstrated for the compounds described in this study.

Due to the close molecular connection between NF-κB and IL-6, we further explored whether actin dynamics are coupled to different aspects of IL-6 signaling. Proteolysis of the IL-6R by ADAM proteases is an important regulatory step in IL-6 biology [20], and shedding of two other ADAM substrates is regulated by interactions with actin. One example is L-selectin/CD62L [24,25], a cell adhesion molecule mostly expressed on leukocytes, that not only regulates actin polymerisation via Rac2 [26], but whose proteolysis depends on the interaction with the actin cytoskeleton via ezrin-radixin-moesin proteins [27]. The second example is Tim3, a protein expressed on T cells, whose proteolysis by ADAM proteases was also dependent on actin interaction [28]. In contrast, we did not find evidence that perturbation of the actin cytoskeleton influences ADAM10-mediated proteolysis of the IL-6R.

Interestingly, we found significant and reproducible reduction in STAT3 phosphorylation in response to IL-6 stimulation when Hep3B cells were pre-treated with actin-modulating compounds. Although the exact mechanism has not been experimentally explored in this study, a previous study showed a physical interaction between actin filaments and JAK2 and STAT3 [29]. This interaction regulated their phosphorylation and would be disrupted when we treated cells with substances like cytochalasin and jasplakinolide that result in perturbation of the actin cytoskeleton. Importantly, both stabilizing and depolymerizing agents resulted in reduced STAT3 phosphorylation, underlining that actin dynamics are required for the observed effects.

Interestingly, the same was not observed in HepG2 cells, and while the reason for these differences are not known yet, previous studies have already demonstrated that there are differences in intracellular signal cascades of both cell lines [30]. Whether these observations also hold true for other cell types that respond to IL-6 classic signaling, e.g., T cell lines, B cell lines or monocytic cell lines, has still to be investigated.

## 4. Materials and Methods

### 4.1. Cells Lines

Hep3B cells were obtained from DSMZ (German Collection of Microorganisms and Cell Cultures, Braunschweig, Germany). HepG2 cells were kindly provided from the Department of Pathology of the Medical University Graz (Austria). Both cell lines were grown in DMEM medium (Gibco/Thermo Fisher Scientific, Waltham, MA, USA), supplemented with 10% fetal bovine serum (Gibco/Thermo Fisher Scientific, Waltham, MA, USA), penicillin (50 U/mL) and streptomycin (50 µg/mL, both from Gibco/Thermo Fisher Scientific, Waltham, MA, USA). Cells were cultured in a standard incubator with a water-saturated atmosphere at 37 °C and 5% CO_2_.

### 4.2. Antibodies and Proteins

Monoclonal antibodies against Inhibitor of nuclear factor κB (IκBα), phospho-IκBα, Nuclear factor-κB p65 (NF-κB p65), signal transducer and activator of transcription-3 (STAT3), phospho-STAT3, GAPDH and the polyclonal antibody against phospho-NF-κB p65 were purchased from Cell Signaling Technology, Inc. (Danvers, MA, USA). The secondary antibodies anti-rabbit IgG HRP-linked antibody and anti-mouse IgG HRP-linked antibody were obtained from Cell Signaling Technology, Inc. (Danvers, MA, USA), whereas Precision Protein™ Strep Tactin-HRP Conjugate was from Bio-Rad laboratories (Hercules, CA, USA). Cytochalasin B was from ENZO Life Sciences, Inc. (Farmingdale, NY, USA), cytochalasin D from Sigma Aldrich (Saint Louis, MO, USA) and jasplakinolide from EMD Millipore (Darmstadt, Germany). Human IL-6 was produced in house as described previously [31]. Ionomycin was purchased from Thermo Fischer Scientific (Waltham, MA, USA) and recombinant human TNFα from PeproTech, Inc. (Cranbury, NJ, USA).

### 4.3. Stimulation and Lysis of Cells

For stimulation with actin-modulating compounds, cells were counted and 4.0 × 10^5^ Hep3B and 7.5 × 10^5^ HepG2 cells per well were seeded on a 6-well plate in 3 mL DMEM or 6.5 × 10^4^ Hep3B cells per well were seeded on a 12-well plate in 1 mL DMEM. Cell culture plates were left under standard conditions in a humidified incubator overnight. On the following day, cells were washed with serum free DMEM, then fresh serum free DMEM was added and cells were stimulated with the indicated amounts of actin-modulating compounds for 15 or 120 min. Depending on the experiment, cells were treated with IL-6 (10 ng/mL) or ionomycin (1 µM) for 15 min or 1 h, respectively, before harvesting. Hep3B cells were seeded and stimulated with TNFα (100 U/mL) for 30 min 24 h, 48 h and 72 h after seeding. Cells were scraped on ice and the pellet was lysed in appropriate volume (e.g., 80 µL/well for 12-well plate and 100 µL/well for a 6-well plate) of Promocell lysis buffer (1 mL Tris HCL 1M pH 7.5; 820 mg NaCl; 1ml Triton × 100; 1 mL 0.5 M EDTA (pH 8.0)). Before usage, 10 µL protease inhibitor cocktail (Sigma-Aldrich, Saint Louis, MO, USA) and 10 µL phosphatase inhibitor cocktail (Sigma-Aldrich, Saint Louis, MO, USA) were added to 1 mL lysis buffer.

### 4.4. Western Blotting and Densitometric Analysis

Protein concentrations were determined using Bio-Rad DC™ Protein Assay (Bio-Rad laboratories, Hercules, CA, USA). A total of 30 to 35 µg of total protein were separated on a 10% or 12% SDS gel and afterwards transferred onto a nitrocellulose membrane by semi-dry blotting. Membranes were blocked with blocking buffer (5% BSA [Carl Roth GmbH + Co. KG, Karlsruhe, Germany] in T-BST and 1% NP 40 [Appli Chem GmbH, Darmstadt, Germany]), washed three times with washing buffer (TBST: 100 mL TBS 10×, 900 mL aqua dest., 1 mL Tween 20 [Carl Roth GmbH + Co. KG, Karlsruhe, Germany]) and incubated overnight at 4 °C with antibodies against NF-κB p65, pNF-κB p65, IκBα, pIκBα, STAT3, pSTAT3 or GAPDH diluted in 5% BSA in TBST following the instructions of the manufacturer. After three washing steps with TBST, HRP-conjugated secondary antibodies, diluted in 5% milk powder in TBST, were added to the membranes and incubated for 1 h at room temperature. Following three washing steps and incubation with Immobilon Western HRP Substrate (EMD Millipore, Darmstadt, Germany), detection of chemiluminescent blots was achieved using a western blot imaging system called the Fluor Chem E System (Protein Simple, San Jose, CA, USA). Protein bands were visualized and quantified using Image Studio version 5.2 (LI-COR Biosciences, Lincoln, NE, USA).

### 4.5. ELISA

96-well ELISA plates were coated with 50 µL capture antibodies against sIL-6R or IL-6 (Duo Set^®^ Human sIL-6Rα and Human IL-6 Duo Set ELISA kits from R&D Systems, Minneapolis, MN, USA), diluted in PBS according to manufacturer’s instructions, and incubated overnight at room temperature. On the following day, non-bound capture antibodies were aspirated and wells were washed twice with 300 µL PBST (1 tablet PBS-Tween^®^ Tablets solved in 1000 mL aqua dest). The plate was blocked with 1% BSA in PBS at room temperature for 1 h. After aspiration/wash step was repeated, 50 µL respectively from standards and samples were added in each well in triplicate and incubated for 2 h at room temperature. Wells were washed afterwards twice and 50 µL detection antibodies, diluted in 1% BSA in PBS, were added per well and incubated for 1 h. Following this, the wash step was repeated and 60 µL streptavidin-coupled horseradish peroxidase (streptavidin-HRP) (R&D systems, Minneapolis, MN, USA), diluted in 1% BSA in PBS were pipetted into each well. After 20 min incubation and being washed twice, 60 µL BM Blue POD Substrate (Roche Diagnostics, Mannheim, Germany) were added into each well to perform the enzymatic reaction. The optical density was measured using a CLARIO star^®^ Plus microplate reader (BMG Labtech, Ortenberg, Germany), set to 450 nm wavelength.

### 4.6. Presentations of Experimental Data and Statistical Analyses

Quantitative data are expressed as the means ± SD. For all western blots, one representative example is shown. Statistical significance of the intergroup differences was determined using GraphPad Prism with one-way analysis of variance (ANOVA) or two-tailed Student’s unpaired t-test as described in the figure legends.

## Data Availability

The data presented in this study are available within the article.
